# Heparin-induced thrombocytopenia (and autoimmune heparin-induced thrombocytopenia): an illustrious review

**DOI:** 10.1016/j.rpth.2023.102245

**Published:** 2023-11-02

**Authors:** Theodore E. Warkentin

**Affiliations:** 1 Department of Pathology and Molecular Medicine, McMaster University, Hamilton, Ontario, Canada; 2 Department of Medicine, McMaster University, Hamilton, Ontario, Canada

A key purpose of medical literature is to inform and educate clinicians on new data, observations, treatments, outcomes, and so forth. The rapid acceleration of knowledge, manifesting through the burgeoning medical literature, increases demand for organized “review articles.” There is a growing trend for such reviews to use visual illustrations, including graphical abstracts and even “illustrated reviews,” in which information transfer relies predominantly on illustrations, with minimal text. *Research and Practice in Thrombosis and Haemostasis* is at the forefront of this knowledge translation shift to illustrated reviews [1].

May et al. [2] have prepared such a review of the complex and counterintuitive adverse drug reaction, entitled “Heparin-induced thrombocytopenia: an illustrated review.” Their work is beautiful, comprehensive, grounded in both established and new scientific concepts in heparin-induced thrombocytopenia (HIT), and deserving of the appellation, an “illustrious” illustrated review. The authors tackle major HIT issues, such as incidence, pathophysiology, diagnosis (divided into clinical and laboratory evaluation and their integration), HIT management (from the perspective of the sequential phases of HIT), and selection of nonheparin anticoagulation, as well as specialized topics, such as performing heart surgery during acute or previous HIT and so-called “autoimmune HIT” (aHIT). Each of these aspects of HIT has been translated into a visually appealing and effective presentation.

The severe subset of HIT known as aHIT [3] is responsible for considerable morbidity and mortality, warranting further attention. Capsule 1—adapted from the work of May et al. [2]—lists 6 aHIT disorders and presents pathogenesis. The definition of aHIT remains in flux, and some classifications of anti-platelet factor 4 (PF4) disorders [4] define aHIT as requiring a triggering exposure to heparin (or low-molecular-weight heparin or fondaparinux); thus, in my slightly altered version of the May et al. [2] capsule, I have separated out “spontaneous HIT” (SpHIT), which by definition has a nonheparin trigger [5], such as knee replacement surgery or infection (sometimes, no precipitant is identified).

**Capsule 1:**
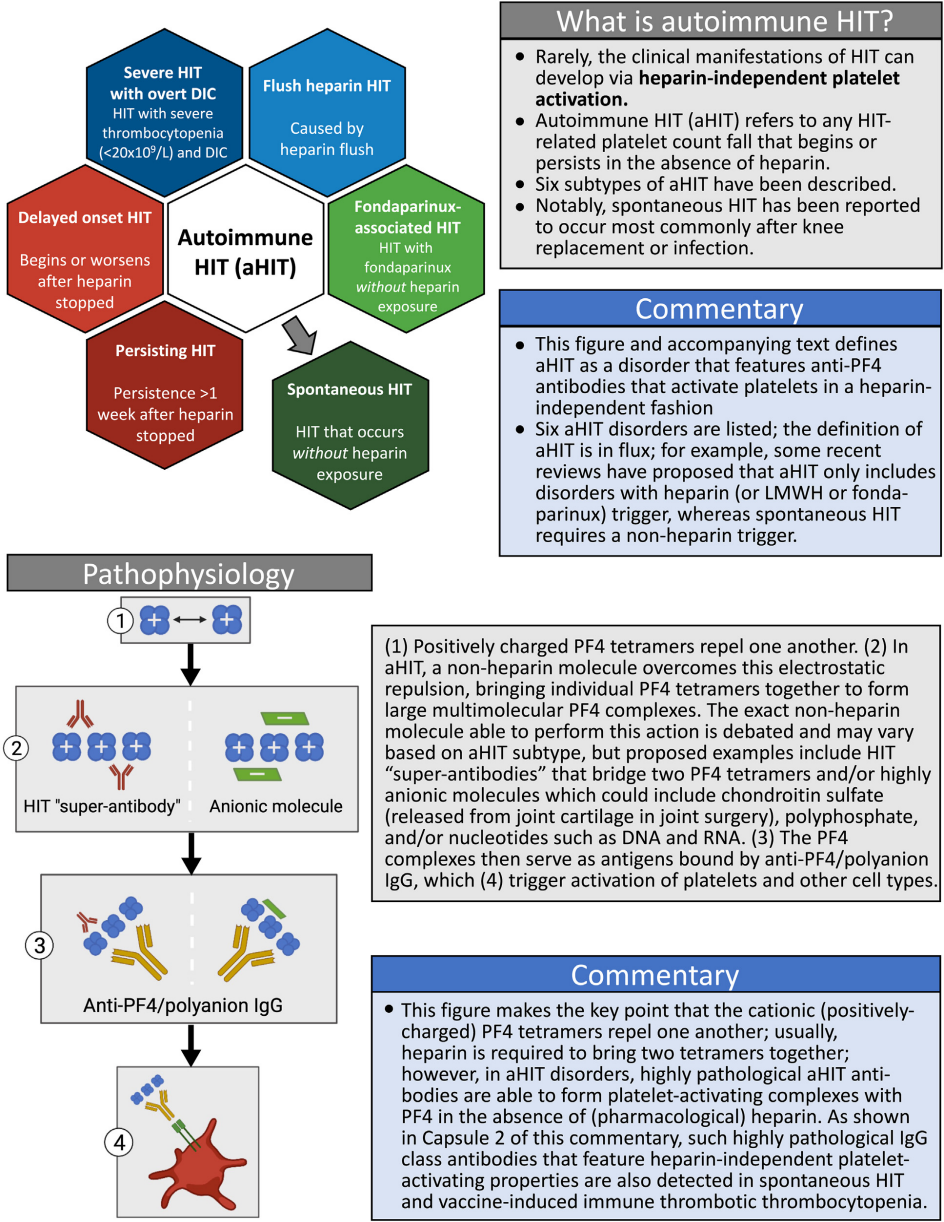
Autoimmune heparin-induced thrombocytopenia (aHIT): definition and pathogenesis. aHIT refers to a disorder triggered by heparin whereas spontaneous heparin-induced thrombocytopenia (SpHIT) requires a nonheparin trigger. aHIT pathogenesis primarily reflects highly pathological aHIT antibodies that can bring PF4 tetramers together in the absence of (pharmacological) heparin. DIC, disseminated intravascular coagulation; IgG, immunoglobulin G; LMWH, low-molecular-weight heparin; PF4, platelet factor 4.

The lower portion of Capsule 1 illustrates the unusual nature of aHIT antibodies, highlighting their heparin-independent platelet-activating properties. Two explanations are presented: (a) HIT “superantibodies” that form immunoglobulin G–PF4 complexes in the absence of polyanion or (b) atypical HIT antibodies that can form complexes with PF4 with polyanions already found within platelets (eg, polyphosphates, chondroitin sulfate, and RNA), without any requirement for (pharmacological) heparin. In the corresponding version of Capsule 1 found in the article by May et al. [2] is the statement, “functional assay may show platelet activation in the absence of heparin, but a non-heparin control is not performed in all laboratories.” Indeed, performing a functional test such as the serotonin-release assay only in the presence of differing heparin concentrations, but not at 0-U/mL heparin (“buffer control”), helps explain the widespread underrecognition of aHIT [6].

As, due to space constraints, May et al. [2] included only a single page discussing aHIT, I have taken this opportunity to pen an editorial commentary that in itself is an illustrated commentary of an illustrated review. In Capsule 2, I summarize the distinction between “classic” HIT—featuring predominantly heparin-dependent antibodies—vis-à-vis aHIT, along with 3 other anti-PF4 disorders that also feature heparin-independent platelet-activating antibodies: the aforementioned SpHIT, vaccine-induced immune thrombotic thrombocytopenia (VITT), and a more recently described nonvaccine-induced VITT-mimicking disorder, “spontaneous VITT” [7]. The capsule further depicts the PF4 tetramer as a “globe,” with duplicated heparin-dependent antigen sites (north and south “poles”) as well as duplicated heparin-independent antigen sites on the “equator” (analogous to the meridians crossing the equator at 0° and 180° longitude) [8]. As a general rule, classic HIT features a moderate degree of thrombocytopenia, moderate thrombotic risk, and rapid platelet count recovery after heparin cessation; in contrast, aHIT and the other anti-PF4 disorders feature more severe thrombocytopenia, very high thrombosis risk (>95%), prolonged thrombocytopenia irrespective of heparin administration, and often overt, decompensated disseminated intravascular coagulation (DIC).

**Capsule 2:**
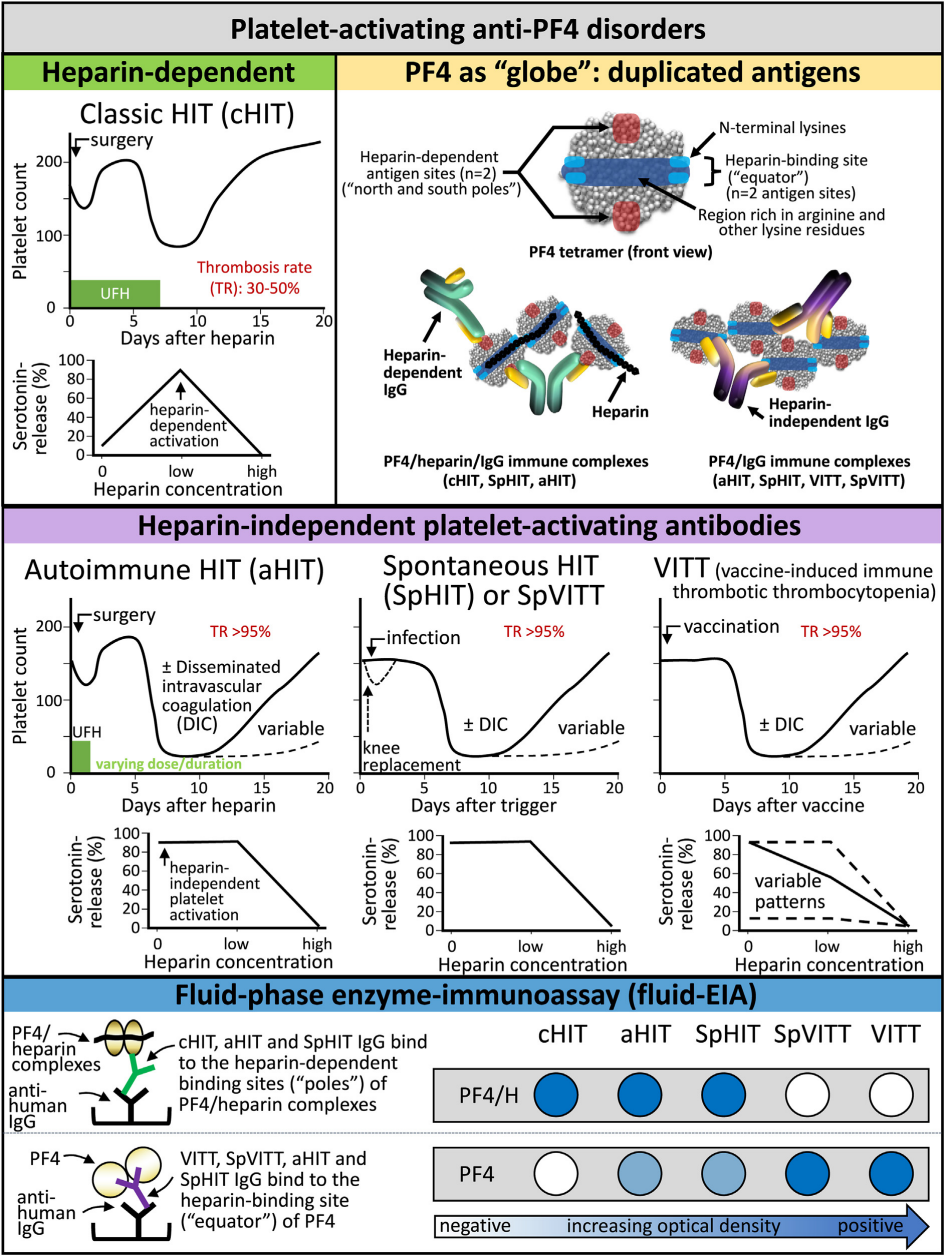
Platelet-activating anti-PF4 disorders. The figure compares the clinical, laboratory, and serologic characteristics of classic heparin-induced thrombocytopenia (cHIT), which features heparin-dependent platelet-activating antibodies, with other anti-PF4 disorders (autoimmune heparin-induced thrombocytopenia [aHIT], spontaneous heparin-induced thrombocytopenia [SpHIT], vaccine-induced immune thrombotic thrombocytopenia [VITT], and spontaneous VITT [SpVITT]), entities with heparin-independent platelet-activating antibodies (aHIT and SpHIT usually have both types of antibodies). SpVITT refers to a disorder that clinically and serologically mimics VITT but does not have an adenovirus vector vaccine trigger (however, a trigger of SpVITT appears to be adenovirus infection itself). The concept of PF4 as a “globe” depicts duplicated heparin-dependent and heparin-independent antigen sites on the “poles” and “equator,” respectively. Representative reaction profiles in the serotonin-release assay and fluid-phase enzyme immunoassay (EIA) are also shown. DIC, disseminated intravascular coagulation; HIT, heparin-induced thrombocytopenia; IgG, immunoglobulin G; PF4, platelet factor 4; TR, thrombosis rate; UFH, unfractionated heparin.

Capsule 3 discusses my editorialist perspective regarding treatment considerations for these atypical anti-PF4 disorders. First, stopping heparin (if being given) has no beneficial effects (after all, these are heparin-independent platelet-activating antibodies), so high-dose intravenous immune globulin becomes a key treatment to de-escalate hypercoagulability through competitive inhibition of platelet FcγIIa receptor–mediated platelet activation [[9], [10], [11]]. Second, anticoagulation with anti–factor Xa action has certain advantages over direct thrombin inhibitors (DTIs) in this situation [12]. For example, the frequent occurrence of concomitant DIC—with resulting prolongation of the activated partial thromboplastin time (aPTT)—can result in systematic underdosing of DTI therapy when monitoring with this global coagulation test (“aPTT confounding” [13]). Furthermore, DTIs impair thrombin-induced activation of protein C [14,15] and could paradoxically contribute to microthrombosis in some patients with severe DIC (indeed, as similarly paradoxical microthrombosis-promoting effects of the vitamin K antagonist, warfarin, was reported [16] in HIT over 20 years ago). For some patients with DIC in the setting of HIT (or SpHIT, spontaneous VITT, or VITT), progression to microvascular thrombosis is a theoretical—although perhaps real [17,18]—risk with DTI therapy.

**Capsule 3:**
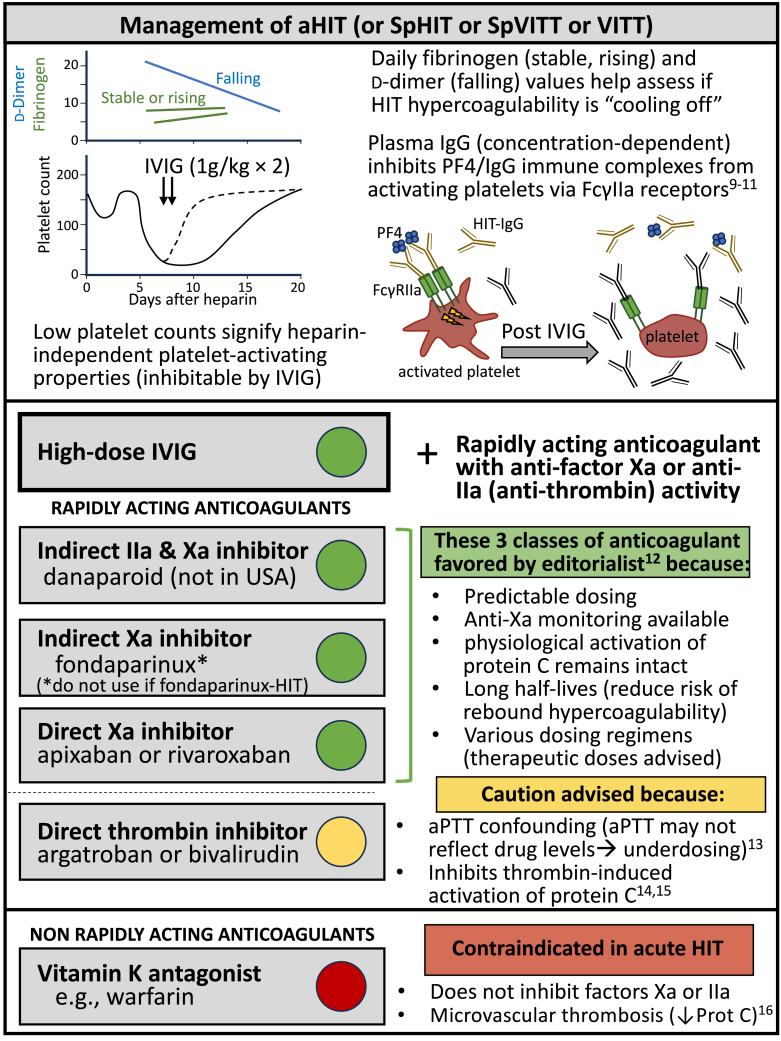
Management of anti-PF4 disorders featuring heparin-independent platelet-activating properties (autoimmune heparin-induced thrombocytopenia [aHIT], spontaneous heparin-induced thrombocytopenia [SpHIT], vaccine-induced immune thrombotic thrombocytopenia [VITT], and spontaneous VITT [SpVITT]). Suggested treatment approaches include daily laboratory monitoring (platelet counts, fibrinogen, D-dimer), high-dose intravenous immunoglobulin (IVIG), and factor Xa inhibiting anticoagulation. aPTT, activated partial thromboplastin time; FcγRIIa, FcγIIa receptor; HIT, heparin-induced thrombocytopenia; IgG, immunoglobulin G; IIa, factor IIa (thrombin); PF4, platelet factor 4; Xa, factor Xa.

There is a large body of literature [19,20] supporting visual communication as an effective tool in scientific/medical knowledge transfer. The arts are known to be powerful, accessible forms of communication, with potential to transfer knowledge by stimulating interest and developing connections. Visual narrative storytelling, such as the illustrated review of May et al. [2], may very well help bridge the gap between research and practice in this area of thrombosis and hemostasis.
